# Incidence and causes of maternal near-miss in selected hospitals of Addis Ababa, Ethiopia

**DOI:** 10.1371/journal.pone.0179013

**Published:** 2017-06-06

**Authors:** Ewnetu Firdawek Liyew, Alemayehu Worku Yalew, Mesganaw Fantahun Afework, Birgitta Essén

**Affiliations:** 1 Department of Nursing, College of Medicine and Health Sciences, Arba Minch University, Arba Minch, Ethiopia; 2 Department of Preventive Medicine, School of Public Health, Addis Ababa University, Addis Ababa, Ethiopia; 3 Department of Reproductive Health and Health Service Management, School of Public Health, Addis Ababa University, Addis Ababa, Ethiopia; 4 Department of Women’s and Children’s Health, International Maternal and Child Health, Uppsala University, Uppsala, Sweden; Federal University of Sergipe, BRAZIL

## Abstract

**Background:**

Because maternal mortality is a rare event, it is important to study maternal near-miss as a complement to evaluate and improve the quality of obstetric care. Thus, the study was conducted with the aim of assessing the incidence and causes of maternal near-miss.

**Methods:**

A facility-based cross-sectional study was conducted in five selected public hospitals of Addis Ababa, Ethiopia from May 1, 2015 to April 30, 2016. All maternal near-miss cases admitted to the selected hospitals during the study period were prospectively recruited. World Health Organization criteria were used to identify maternal near-miss cases. The number of maternal near-miss cases over one year per 1000 live births occurring during the same year was calculated to determine the incidence of maternal near-miss. Underlying and contributing causes of maternal near-miss were documented from each participant’s record.

**Results:**

During the one-year period, there were a total of 238 maternal near-miss cases and 29,697 live births in all participating hospitals, which provides a maternal near-miss incidence ratio of 8.01 per 1000 live births. The underlying causes of the majority of maternal near-miss cases were hypertensive disorders and obstetric hemorrhage. Anemia was the major contributing cause reported for maternal near-miss. Most of the maternal near-miss cases occurred before the women’s arrival at the participating hospitals.

**Conclusion:**

The study demonstrated a lower maternal near-miss incidence ratio compared to previous country-level studies. The majority of the near-miss cases occurred before the women’s arrival at the participating hospitals, which underscores the importance of improving pre-hospital barriers. Efforts made toward improvement in the management of life-threatening obstetric complications could reduce the occurrence of maternal near-miss problems that occur during hospitalization.

## Introduction

The improvement of maternal health has made slow progress in most of the sub-Saharan African countries [1]. According to the World Health Organization (WHO), the United Nations Children's Fund (UNICEF), the United Nations Population Fund (UNFPA) and the World Bank (2014) estimate, globally, 289,000 maternal deaths occurred in 2013, with the highest burden being in sub-Saharan African countries [1]. Despite the high number of maternal deaths in many of the institutions within these countries, the absolute number for each center classifies these events as rare, which leads to a reduced level of power to allow the studies to investigate the potential risk factors. Thus, in this situation, severe acute maternal morbidity or maternal near-miss could serve as a surrogate for maternal death to evaluate the quality of obstetric care in particular health institutions. A maternal near-miss event or severe acute maternal morbidity is currently defined by the WHO as ‘a woman who nearly died but survived a complication that occurred during pregnancy, childbirth or within 42 days of termination of pregnancy’ [2–5].

Because there were no uniform criteria for the identification of near-miss cases and no standard definition for maternal near-miss until 2009, a heterogeneous estimate of rates was observed in different published literatures around the world. For instance, the rate ranged between 0.14% and 0.75% in some of the high-income countries [6–13], it ranged between 1.5% and 7.7% in some of middle- income countries [14–18], and, in sub-Saharan African countries, it ranged between 2.21% and 12% [19–22]. In Ethiopia, the prevalence has reached as high as 7.9% [23].

Worldwide, hypertensive diseases of pregnancy, obstetric hemorrhage, sepsis, anemia and obstructed labor/dystocia have been identified as the major causes of maternal near-miss [8,14,24–26].

Ethiopia, as in many developing countries, has a high rate of maternal mortality. According to the 2011 Ethiopian Demographic and Health Survey (EDHS), the maternal mortality ratio (MMR) of the country was 676 per 100,000 live births, and the 2016 EDHS recorded 412 deaths per 100,000 live births [27,28]. An estimated 2.9 million women give birth every year in Ethiopia, and only 26.2% of them deliver in a health facility [28,29]. According to the recent 2016 EDHS report, the percentage of women who received Antenatal care (ANC), delivery care and a postnatal check-up from a skilled provider was 62.4%, 27.7% and 16.5%, respectively [28].

The Federal Ministry of Health of Ethiopia is striving to reduce the rate of maternal mortality of the country. Actions taken so far include organizing and mobilizing the Health Development Army at all levels to promote behavioral change, the distribution of ambulances to all districts of Ethiopia, the provision of free maternity services at different health care levels, the training of human resources and equitable placement of health professionals in health facilities, and the provision of adequate drugs, medical supplies, and equipment [30]. Despite all of these efforts, the maternal mortality rate of the country remains high. Therefore, there is a need to assess the magnitude and possible causes that contribute to maternal mortality. However, because maternal near-miss is a rare event, and because it follows a similar pathway to maternal deaths, there is a benefit to including a larger number of cases for analysis, as research related to maternal near-miss is crucial when examining the quality of obstetric care [2,4,31]. In Ethiopia, maternal near-miss complications are common and are estimated to be around 12 times more frequent than maternal deaths [23]. However, the incidence and causes of near-miss events are not well documented. Thus, this study aimed to assess the incidence and causes of maternal near-miss.

## Methods

### Study settings and period

**Fig 1:**
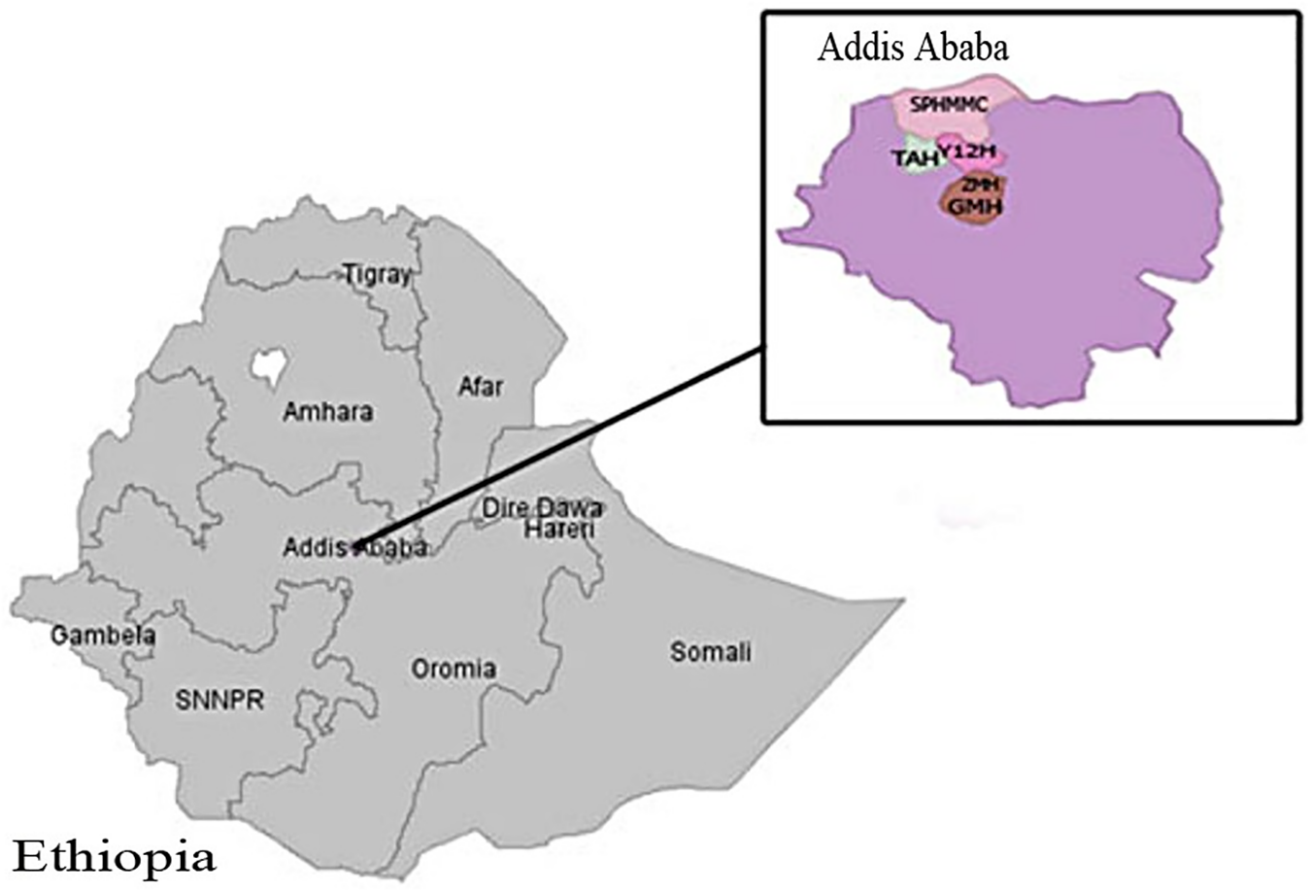
Location map of the study area in Addis Ababa, Ethiopia. SPHMC (St. Paul’s Hospital Millennium Medical College), TAH (Tikur Anbessa Hospital), Y12H (Yekatit 12 Hospital), ZMH (Zewditu Memorial Hospital) and GMH (Gandhi Memorial Hospital).

The study was conducted in five selected public hospitals of Addis Ababa, Ethiopia from May 1, 2015 to April 30, 2016. The hospitals were selected based on the number of deliveries they managed per year. Because most critical maternal cases are referred to a hospital known to provide better care, the presence of an Intensive Care Unit (ICU), maternity ward, blood transfusion service and facilities for caesarean section (CS) were also considered in the selection of hospitals. Hence, Tikur Anbessa, St. Paul’s Hospital Millennium Medical College, Zewditu Memorial, Yekatit 12, and Gandhi Memorial Hospitals were selected for the current study. Tikur Anbessa Hospital is the largest referral and teaching hospital in Ethiopia and is operated under the Ministry of Education of Ethiopia. St. Paul’s Hospital Millennium Medical College is the largest referral and teaching hospital among those operated under the Federal Ministry of Health. However, the Gandhi Memorial, Yekatit 12 and Zewditu Memorial Hospitals were among the six governmental referral and teaching hospitals that are managed under the Addis Ababa Administrative Health Office. Together, the five hospitals were responsible for a total of 29,697 live birth deliveries during the year in which this study took place. Apart from Tikur Anbessa Hospital, which received very critical cases from different part of Ethiopia, the hospitals are comparable in terms of the patients they receive for care and treatment (Fig 1).

### Study design

A facility-based cross-sectional study design was used to address the objective of the current study.

### Identification of cases

All women admitted to the participating hospitals during the study period for the treatment of pregnancy-related complications (such as ectopic pregnancy or abortion), having delivered, or within 42 days of termination of pregnancy, and who fulfilled at least one of the conditions stated in the WHO criteria (S1 Table) [5] were included. Depending on when the near-miss occurred, maternal near-miss cases were further categorized into two groups. Women who were assessed as being in critical condition on arrival to a hospital were classified as near-miss before arrival. However, if the near-miss occurred during hospitalization, it was classified as near-miss after arrival.

### Sample size determination

The sample size was determined by a single population proportion formula by assuming the prevalence of maternal near-miss in Ethiopia to be 7.9% [23]. Considering a 1% margin of error, a 95% confidence interval (CI) and a 10% non-response rate, a minimum of 2795 live births were calculated to be the appropriate sample size for this study. However, during the year of the study, a 10 times larger number of live births than the number required was obtained in the five hospitals (29,697 live births), and we decided to include the entire period of one year to increase the precision of the study.

### Data collection

Women who experienced a maternal near-miss event during pregnancy, delivery or the postpartum period were identified prospectively by well-trained midwives and nurses in each hospital. Data relating to the most important variables were abstracted from the medical record of the participants using the WHO data abstraction tool, with some modifications [5]. The data were collected from the Delivery Ward, Obstetrics and Gynecology Ward, ICU, and Emergency Gynecology Outpatient Department of each hospital. For each maternal near-miss case, only one underlying cause was identified as per the WHO International Statistical Classification of Diseases and Related Health Problems (ICD). According to the ICD, the underlying cause is the disease or injury which initiated the sequence of events leading directly to death [32]. Because the same classification is used for both maternal death and maternal near-miss [33], the classifications used for maternal near-miss were the same as those listed in the ICD for maternal mortality [34]. However, all possible contributing causes were considered. Information regarding whether the near-miss was present before arrival or developed during hospitalization was also collected in order to determine the place where the near-miss occurred. Data on the total number of live births occurring over one year for each hospital were extracted from the Health Management Information System (HMIS) report of each hospital.

### Data processing and management

The supervisors in all participating hospitals were responsible for checking the completeness of the information. The enumerators filled in the date and signed each questionnaire, which was later checked, edited and signed by the supervisors regularly at each hospital. The data that were collected using hard copies were kept in a locked cabinet by each supervisor until gathered by the principal investigator during supervision. Following this, the data were entered into Epi Info 7 software, and transported to SPSS version 20 and Open Epi computer software for final analysis.

### Data analysis

The total incidence of maternal near-miss in the hospitals involved in this study was calculated using the maternal near-miss incidence ratio (MNMIR) formula. This was calculated by dividing the number of maternal near-miss cases during one year by the total number of live births during the same year. The incidence ratio in each hospital was also calculated with a 95% CI. In addition, hospital access indicators, such as the number of women with a maternal near-miss condition before arrival at the hospital, were calculated. Intra-hospital care indicators, such as the number of women with near-miss who developed conditions in the hospital, were also calculated. In order to determine the underlying and contributory causes of maternal near-miss, a descriptive frequency for each cause was calculated. The total number and frequency of each cause for all hospitals involved were calculated separately. The causes were categorized into underlying and contributory as per the WHO recommendation [5]. A descriptive frequency of the type of organ dysfunction present in maternal near-miss cases was also calculated.

### Data quality assurance

In order to maintain the quality of data, intensive training was given to data collectors and supervisors. All health care workers working in the maternity ward of each participating hospital were also sensitized to the issue so that they would inform the enumerators when they suspected a near-miss case. In addition, inclusion criteria for maternal near-miss were printed and posted on the wall of each ward at all participating hospitals. The data collectors made a daily visit to the Delivery Ward, Obstetrics and Gynecology Ward, ICU, and Emergency Gynecology Outpatient Department to check for potential cases. The data collectors were given training to standardize methods and ensure consistency of data collection. One hospital supervisor, who was responsible for the overall quality of the data, was appointed at each participating hospital. There was frequent supervision of the included hospitals by the principal investigator. The standardized data abstraction form developed by the WHO [5] was used to abstract pertinent information. The questionnaires were also first pre-tested in the participating hospitals to verify the appropriateness of the tool. The standardized WHO criteria were used to identify maternal near-miss cases. Hence, all the above procedures contributed greatly to obtaining quality data.

### Ethics statement

Acceptable ethical standards were strictly adhered to throughout the study process. The study was first approved by the Institutional Review Board of the College of Health sciences, Addis Ababa University (Protocol number: 058/14/SPH, Date: January 2015). It was also approved by the Ethical Review Committee of each hospital. Adequate explanation about the purpose of the study and a letter of support was given to all concerned bodies. For studies that are not clinical trials that involve invasive procedures, taking verbal consent is the standard requirement of the Institutional Review Board of Addis Ababa University. Hence, verbal consent was taken to abstract pertinent information from the participant’s record. The anonymity of the participants was respected via the use of codes rather than the name of the participant. The names of the participants were not reported in the findings of the study to ensure confidentiality.

## Results

### Incidence of maternal near-miss

During the one-year period, a total of 238 maternal near-miss cases and 29,697 live births were reported in total for all participating hospitals, which produced a total maternal near-miss incidence ratio of 8.01 per 1000 live births (95% CI; 7.06–9.09).

The highest proportion of cases was reported from Tikur Anbessa hospital (30.7%), followed by St. Paul Millennium Medical College (23.1%), and the lowest proportion was observed at Zewditu Memorial Hospital (Table 1).

**Table 1:** Incidence of maternal near-miss in five selected public hospitals of Addis Ababa, Ethiopia from May 1, 2015 to April 30, 2016.

Name of Hospital	Near-miss cases *(n)*	Percentage (%)	Total live births in one year	*MNMIR per 1000 live births (95% CI)
Tikur Anbessa	73	30.7	4632	15.8 (12.6–19.8)
St. Paul Millennium Medical College	55	23.1	9079	6.06 (4.66–7.88)
Gandhi Memorial	39	16.4	7091	5.49 (4.02–7.51)
Zewditu Memorial	20	8.4	4610	4.34 (2.81–6.69)
Yekatit 12	51	21.4	4285	11.9 (9.06–15.61)
Total	238	100	29,697	8.01 (7.06–9.09)

* MNMIR represents maternal near-miss incidence ratio

### Characteristics of women with maternal near-miss

The majority (88.2%) of maternal near-miss cases were referred from other health facilities and an ambulance was used by most of the mothers as a means of transport to the study hospitals. A significant number (68.5%) of maternal near-misses occurred before arrival at the participating hospitals (Table 2).

**Table 2:** Characteristics of women with maternal near-miss in five selected public hospitals of Addis Ababa, Ethiopia from May 1, 2015 to April 30, 2016.

Variable	Number	percent
**Admission mode (*n* = 238)**		
Self-referred	28	11.8
Referred from other facility	210	88.2
**Means of transport used (*n* = 238)**		
Ambulance	181	76.1
Public transport	38	16
Personal vehicle	15	6.3
Others	4	1.7
**When did the near-miss occur? (*n* = 238)**		
Before arrival	163	68.5
During hospitalization	75	31.5

### Organ dysfunction in maternal near-miss cases

**Fig 2:**
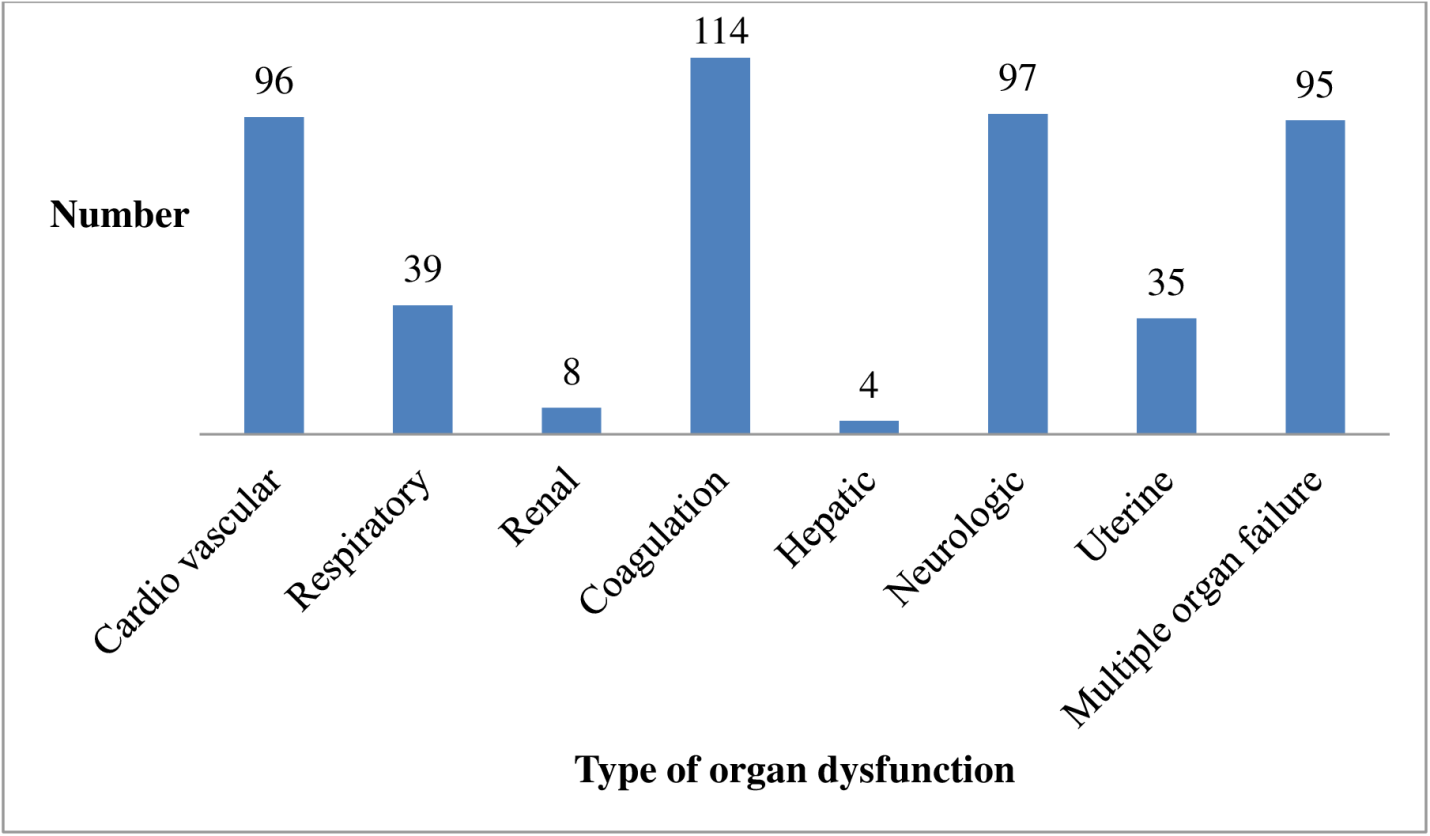
Organ dysfunction in maternal near-miss cases in five selected public hospitals of Addis Ababa, Ethiopia, May 2015 to April 30, 2016.

The number of major organ dysfunctions seen in the majority of maternal near-miss cases were Coagulation/Hematological at 114 (47.9%), followed by Neurologic at 97 (40.8%), and Cardiovascular at 96 (40.3%). Hepatic dysfunction was the least-reported organ dysfunction in audited maternal near-miss cases. Around 95 (39.9%) of the cases manifested multiple organ failure (Fig 2).

### Underlying and contributory causes of maternal near-miss

**Fig 3:**
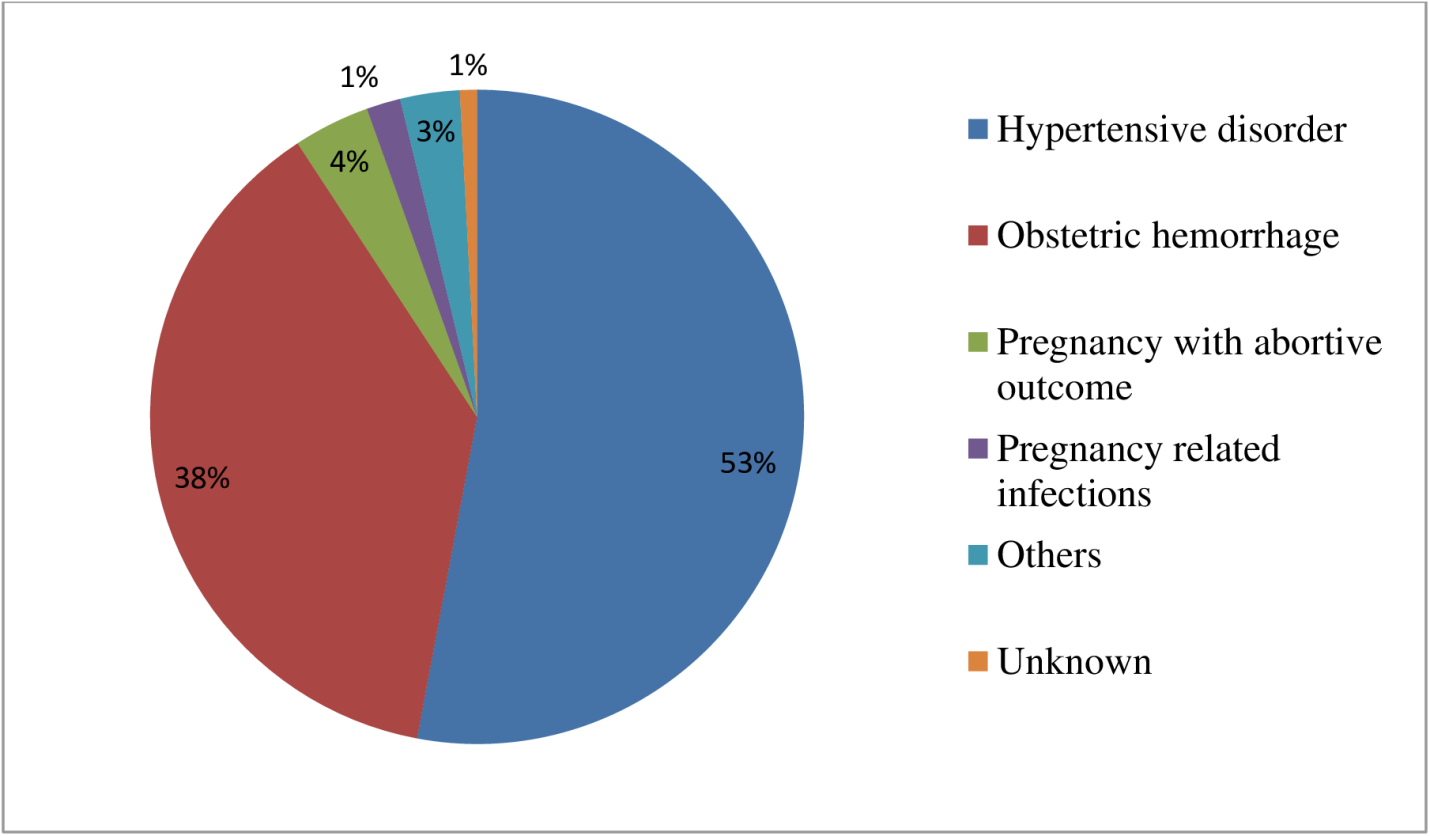
Underlying causes of maternal near-miss in five selected public hospitals, Addis Ababa, Ethiopia May1, 2015 to April 30, 2016.

The underlying cause for the majority of maternal near-miss cases was hypertensive disorder (53%), followed by obstetric hemorrhage (38%), pregnancy with abortive outcome (4%), and pregnancy-related infections (1%) (Fig 3).

**Fig 4:**
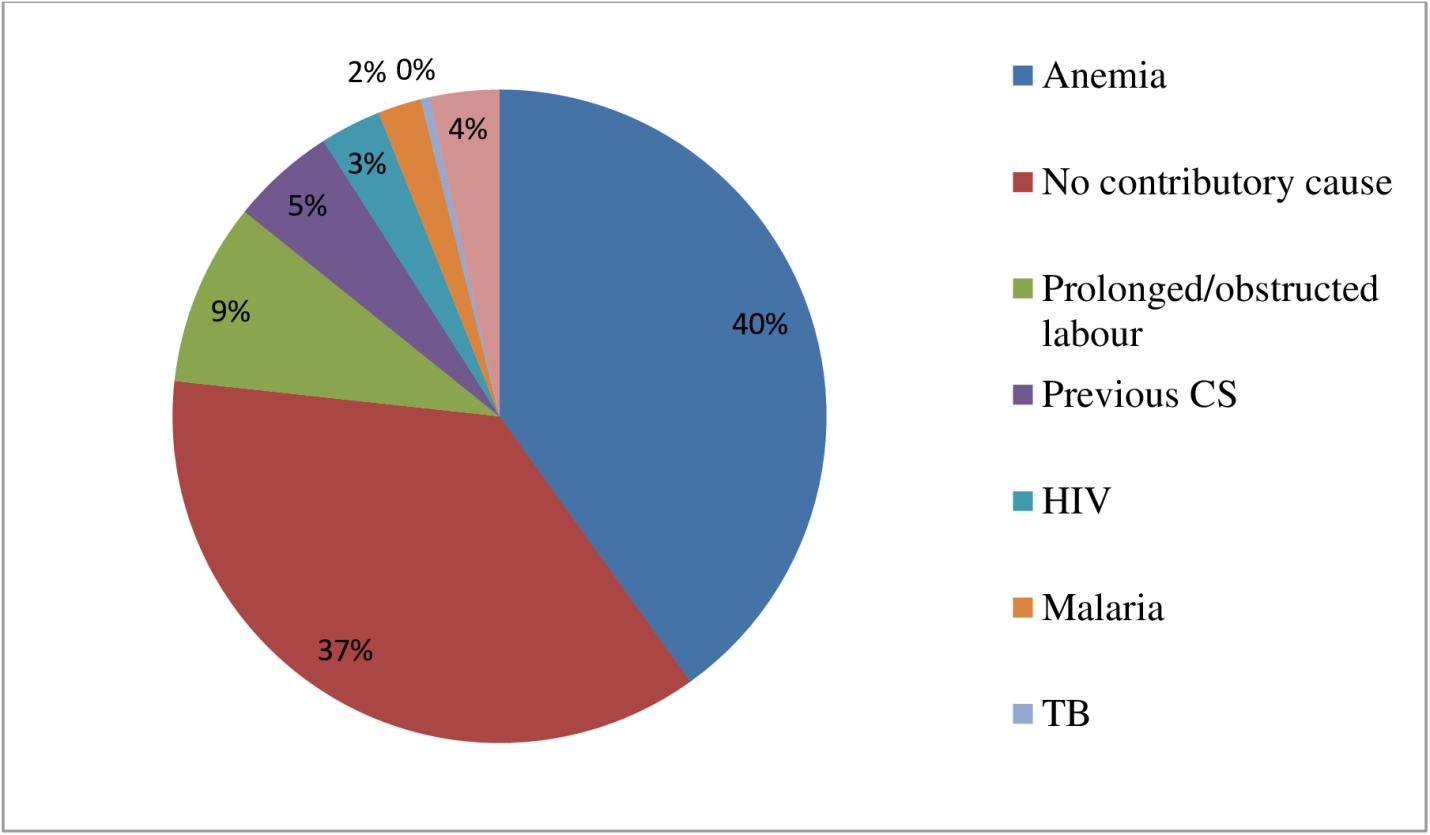
Contributing causes of maternal near-miss in five selected public hospitals, Addis Ababa, Ethiopia May1, 2015 to April 30, 2016.

The major contributing causes of maternal near-miss reported were anemia (40%) followed by prolonged/obstructed labor (9%). Around 37% of maternal near-miss cases did not show any form of contributing causes (Fig 4).

## Discussion

### Incidence of maternal near-miss

During the one-year period of the study, the incidence of maternal near-miss was 8.01 per 1000 live births in all participating hospitals. Previous studies in Ethiopia have documented a prevalence rate of 101 per 1000 deliveries [26] and 78.9 per 1000 live births [23]. Surprisingly, our finding is considerably lower than that given in previous reports and the observed variation could be a result of disparity in the case definitions used by the researchers, the study design used, and the time at which the study was conducted. We used the newly developed WHO criteria, which are very stringent and would identify only very critical cases. However, previous studies used disease-based criteria, which were less stringent than the WHO criteria for identifying maternal near-miss cases. Thus, had the previous studies employed the WHO criteria, they might have ended up reporting more cases. Data quality issues and the limitations of the secondary data obtained in previous studies might also be an alternative explanation for the observed difference. Nevertheless, when we compare our findings with other studies that used the newly developed WHO criteria, the incidence was lower than some other sub-Saharan African countries such as Ghana and Tanzania [19,35]. However, our results are comparable with studies conducted in Rwanda and Uganda, where they reported an incidence rate of 8 per 1000 live births and 8.42 per 1000 live births, respectively [36,37].

Among the five participating hospitals, a higher incidence of maternal near-miss was observed in Tikur Anbesa Hospital (15.76 per 1000 live births). Because that hospital is the major referral hospital in Ethiopia, the possibility of obtaining severely critical cases from different parts of the country and from Addis Ababa is higher.

### Causes of maternal near miss

The leading underlying cause of maternal near-miss in our study was hypertensive disorder (eclampsia and pre-eclampsia), followed by obstetric hemorrhage. This finding is compatible with most studies from high and middle-income countries [8,9,38,39]. The study finding was also in line with studies conducted in other African countries [20,21,25]. A previous study in Ethiopia also reported hypertensive disorder as the primary cause, and obstetric hemorrhage as the second leading cause of maternal near-miss [23]. High percentages of hypertensive disorder and obstetric hemorrhage might be indicative of some form of delay in managing obstetric complications by the facility staff.

Pregnancy-related infection was the least mentioned cause of maternal near-miss in our study and was also reported to be the least likely cause in most of the studies completed in developed countries. The rate was also much lower as compared to some of the other African countries that had been studied [20–22,25]. The lower percentage of infection as a cause of maternal near-miss could be explained by the presence of early management of the cases with appropriate antibiotics at each health facility.

Anemia was the major contributory cause of maternal near-miss in our study. This finding is also comparable with studies from some middle-income countries such as Iraq, India, and Pakistan [40–42]. This finding was also in line with the studies from some African countries such as Ghana [19]. The presence of anemia in women can be attributed to nutritional and iron deficiency during pregnancy. It could also result from the presence of previous malaria. Hence, there is a need to deeply assess the causes of anemia in women with a maternal near-miss case to determine the most appropriate action.

The majority of maternal near-miss cases have already occurred on the women’s arrival at the participating hospitals, a finding which is in line with studies from most developing countries. For example, in Bolivia, Mozambique and Somaliland, 74%, 70.7% and 74.2% of the near-miss cases, respectively, were in a critical state upon arrival at the health facilities, implying the need to focus on existing pre-hospital barriers [43–45]. However, near-miss cases that develop during hospitalization can help to measure the quality of obstetric care provided within the health facilities. In Iran, for example, sub-optimal obstetric care was found in 75% of the near-miss cases [46]. The occurrence of maternal near-miss after receiving sub-optimal care following caesarian section has also been reported elsewhere [47]. However, it should be noted that quality of care is not the only possible explanation for near-miss events occurring during hospitalization. Cases that occurred after admission could also be related to the severity of the cases.

This study has many strengths. The study is the first of its kind in Ethiopia to document the incidence and causes of maternal near-miss using the newly developed WHO case identification criteria. Prospective case identification was used for a consecutive period of one year. Identifying cases prospectively over a longer period of time enabled us to avoid missing important variables, which is a drawback of most previous retrospective studies. Collecting data over a longer period of time can also help to gather a representative sample. In using the WHO criteria, we were able to determine which of the criteria were mainly applicable to the study hospitals. The use of a standardized WHO data abstraction tool to abstract data was also one of the strengths of the study, which might also have had its own implications for the quality of the study.

However, our study had certain limitations. The follow-up time used by the WHO to define maternal near-miss has a duration of 42 days postpartum. However, because of logistic and feasibility concerns, our follow-up time was limited to only the length of the hospital stay. This might have caused us to underestimate the magnitude of maternal near-miss. The study also failed to abstract all maternal near-miss indicators such as the interventions provided for maternal near-miss women, which are important components of quality of care. The other limitation of the study was that our study was carried out only in public health facilities. Hence, it does not represent cases of maternal near-miss that occur in private health facilities.

## Conclusion

The study demonstrated a lower maternal near-miss incidence ratio compared to previous country-level studies. Underlying and contributory causes of maternal near-miss are still prevalent. Evidence-based interventions designed to optimize the intra-partum management of life-threatening obstetric complications, specifically hypertensive disorders and obstetric hemorrhage, could reduce the occurrence of maternal near-miss problems occurring during hospitalization. The majority of the near-miss cases happened before the women’s arrival at the participating hospitals, which underscores the importance of eliminating the pre-hospital barriers. Hence, further research is recommended to explore those barriers.

## Supporting information

S1 Table Identification criteria of maternal-near miss as used by the WHO, 2009 and 2011. (DOCX)
