# Supplementary material for: Incidence and causes of maternal near-miss in selected hospitals of Addis Ababa, Ethiopia
Source: PLoS One. 2017 Jun 6;12(6):e0179013. doi: 10.1371/journal.pone.0179013 (PMC5460898; doi:10.1371/journal.pone.0179013)
Supplement: S1 Table — (DOCX) [file pone.0179013.s001.docx]

| Dysfunctional system | Clinical criteria | Laboratory markers | Management based proxies |
| --- | --- | --- | --- |
| **Cardiovascular** | shock  Cardiac arrest | severe hypo perfusion  (lactate >5 m mol/l or >45 mg/dl)  severe acidosis (pH <7.1) | Use of continuous vasoactive drugs  Cardio pulmonary resuscitation |
| **Respiratory** | Acute cyanosis  Gasping  severe tachypnea (respiratory rate >40 breaths per minute)  severe bradypnea (respiratory  rate <6 breaths per minute) | severe hypoxemia (O2 saturation <90% for  ≥60 minutes or PAO2/FiO2 <200) | Intubation  and ventilation not related to anesthesia |
| **Renal** | Oliguria non-responsive to fluids or diuretics | severe  acute azotemia (creatinine ≥300 µmol/ml  or ≥3.5 mg/dl) | dialysis for acute renal failure |
| **Coagulation/hematological** | Failure to form clots | severe acute thrombocytopenia (<50 000  platelets/ml) | massive transfusion of blood or red cells (≥5 units) |
| **Hepatic** | Jaundice in the presence of pre-eclampsia | severe acute hyperbilirubinemia  (bilirubin >100 µmol/l or >6.0 mg/dl) |  |
| **Neurological** | Prolonged unconsciousness (lasting  ≥12 hours)/coma (including metabolic  coma), stroke, uncontrollable fits/status epileptics, total paralysis |  |  |
| **Uterine** |  |  | Uterine hemorrhage or infection leading  to hysterectomy |
